# Changes in the gut microbiome community of nonhuman primates following radiation injury

**DOI:** 10.1186/s12866-021-02146-w

**Published:** 2021-03-29

**Authors:** Raj Kalkeri, Kevin Walters, William Van Der Pol, Braden C. McFarland, Nathan Fisher, Fusataka Koide, Casey D. Morrow, Vijay K. Singh

**Affiliations:** 1 Southern Research, Frederick, MD USA; 2 Center for Clinical Translational Sciences, University of Alabama at Birmingham, Birmingham, AL USA; 3 Department of Cell, Developmental and Integrative Biology, University of Alabama at Birmingham, Birmingham, AL USA; 4 Division of Radioprotectants, Department of Pharmacology and Molecular Therapeutics, F. Edward Hébert School of Medicine, Uniformed Services University of the Health Sciences, Bethesda, MD USA; 5 Armed Forces Radiobiology Research Institute, Uniformed Services University of the Health Sciences, Bethesda, MD USA

**Keywords:** Nonhuman primates, Gut microbiome, Irradiation, Microbiome alterations, Radiation injury, Microbiome marker, Diarrhea

## Abstract

**Background:**

Composition and maintenance of the microbiome is vital to gut homeostasis. However, there is limited knowledge regarding the impact of high doses of radiation, which can occur as a result of cancer radiation therapy, nuclear accidents or intentional release of a nuclear or radioactive weapon, on the composition of the gut microbiome. Therefore, we sought to analyze alterations to the gut microbiome of nonhuman primates (NHPs) exposed to high doses of radiation. Fecal samples were collected from 19 NHPs (Chinese rhesus macaques, *Macaca mulatta*) 1 day prior and 1 and 4 days after exposure to 7.4 Gy cobalt-60 gamma-radiation (LD_70–80/60_). The 16S V4 rRNA sequences were extracted from each sample, followed by bioinformatics analysis using the QIIME platform.

**Results:**

Alpha Diversity (Shannon Diversity Index), revealed no major difference between pre- and post-irradiation, whereas Beta diversity analysis showed significant differences in the microbiome after irradiation (day + 4) compared to baseline (pre-irradiation). The Firmicutes/Bacteriodetes ratio, a factor known to be associated with disruption of metabolic homeostasis, decreased from 1.2 to less than 1 post-radiation exposure. *Actinobacillus, Bacteroides, Prevotella (Paraprevotellaceae family) and Veillonella* genera were significantly increased by more than 2-fold and *Acinetobacter* and *Aerococcus* genus were decreased by more than 10-fold post-irradiation. Fifty-two percent (10/19) of animals exposed to radiation demonstrated diarrhea at day 4 post-irradiation. Comparison of microbiome composition of feces from animals with and without diarrhea at day 4 post-irradiation revealed an increase in *Lactobacillus reuteri* associated with diarrhea and a decrease of *Lentisphaerae* and *Verrucomicrobioa* phyla and *Bacteroides* in animals exhibiting diarrhea. Animals with diarrhea at day 4 post-irradiation, had significantly lower levels of Lentisphaere and Verrucomicrobia phyla and Bacteroides genus at baseline before irradiation, suggesting a potential association between the prevalence of microbiomes and differential susceptibility to radiation-induced diarrhea.

**Conclusions:**

Our findings demonstrate that substantial alterations in the microbiome composition of NHPs occur following radiation injury and provide insight into early changes with high-dose, whole-body radiation exposure. Future studies will help identify microbiome biomarkers of radiation exposure and develop effective therapeutic intervention to mitigate the radiation injury.

**Supplementary Information:**

The online version contains supplementary material available at 10.1186/s12866-021-02146-w.

## Background

Whole-body radiation exposure occurs in a variety of settings including cancer radiotherapy [1], industrial accidents, and intentional release of nuclear or radiologic weapons [2]. While moderate to high level exposure to radiation impacts a number of physiologic systems, acute death is often associated with sepsis. This led us to explore the impact of high-dose, whole-body radiation exposure on the fecal microbiome composition of NHPs.

Cancer remains the second leading cause of mortality (ischaemic heart disease is the highest) with 9.6 million mortality worldwide in 2018 [3]. Treatment for almost all types of cancers includes surgery, chemotherapy, and irradiation. Ionizing radiation results in the damage of DNA, which ultimately results in cell death [4]. Additionally, irradiation results in the creation of free radicals that exert detrimental effects on nearby cells affected by the radiation field area [5]. Rapidly dividing cells, including the targeted cancer cells, are especially sensitive to irradiation.

Although ionizing radiation is highly effective at inducing DNA damage in cancer cells, which leads to cell death, nearby healthy cells are also affected [4]. Localized irradiation is possible with most cancers, but for abdominal cancers a broader field area of ionizing radiation is necessary and can cause serious effects on normal tissue including the gastrointestinal tract. In particular, the epithelial cells of the intestines are rapidly dividing healthy cells commonly adversely affected by irradiation. A common side-effect of irradiation is diarrhea, which can negatively affect quality of life as well as radiation injury treatment outcome [4, 6]. Intestinal radiation injury is a significant clinically unmet challenge. It is estimated that more than 300,000 patients receive pelvic or abdominal radiation therapy and 60–80% (180,000 to 240,000) of these show symptoms of acute bowel toxicity. These annual incidences add up to 1.6 million patients with post-irradiation intestinal dysfunction living in US alone [7].

Commensal microbes within the human gastro-intestinal (GI) tract are vital to achieving and maintaining optimal homeostasis. These functions include the basic food breakdown and absorption of food byproducts as well as production of vitamins. Additional functions include prevention of pathogen colonization, and direct interaction with the immune cells of the GI tract [8]. Dysbiosis or significant changes in the composition of the microbiome has been linked to several diseases including diabetes, heart disease, neurological diseases and cancer [9].

Previous studies have shown that ionizing radiation induces significant changes in the microbiome [4, 10]. More recently, the relationship between ionizing radiation and gut microbiome changes has been demonstrated [11–13]. Microbial diversity correlated with radiation enteropathy and higher levels of *Clostridium IV*, *Roseburia*, and *Phascolarctobacterium* were observed in patients with radiation enteropathy. Though the role of specific microbiome alteration in induction of diarrhea needs to be understood, probiotic usage has shown positive effect in preventing radiation-induced diarrhea [14], suggesting that intentional modulation of the gut microbiome may be one means by which radiation-induced enteropathy can be mitigated. Due to the significant impact of radiation-induced impact on disease outcome and quality of life, in depth analyses of how radiation can affect the microbiome and radiation-induced diarrhea is needed. Radiation medical countermeasures for acute radiation syndrome are being developed following the United States Food and Drug Administration Animal Rule [15]. This rule applies to new countermeasures for which conclusive human efficacy investigations under phase 2 and phase 3 clinical trials cannot be performed due to ethical reasons. According to this rule, the FDA can approve new drugs that have been shown to be safe in humans based on well-controlled animal efficacy studies. NHP is the only well-defined animal species acceptable to FDA for such drug approval and also most close to human with 95% sequence homology at the level of DNA [16]. Thus, NHPs are used to support various aspects of countermeasure development including radiation lethality determination, model refinement, radiation injury biomarker identification, drug efficacy, mechanism of action, and omic studies. Due to the evolutionary proximity to humans, NHPs were used in our study to explore the alteration in the gut microbiome composition after radiation exposure.

Radiation exposure effects include diarrhea due to changes in intestinal microbial flora and damage to the intestinal lining that persist after the cessation of radiation [17]. The objective of this study was to understand the profile of changes in the intestinal microbial flora after irradiation to potentially identify biomarkers of radiation-induced diarrhea. In this study we sought to characterize the GI microbiome alterations following whole-body irradiation in NHPs (Chinese rhesus macaques). Similar to what has been attempted by others [12, 18, 19], we also sought to identify specific microbial changes as a biomarker to predict adverse side effects (irradiation-induced diarrhea) following whole-body irradiation in these animals.

## Methods

### Animals

Nineteen healthy Male Rhesus Macaques (*Macaca mulatta, Chinese strain*), weighing 4.0 to 8.0 kg (average of 4.75 kg, with a Range of 4.09 to 5.48 kg) and between 2.5 and 7 years of age (Mean of 4.7 years with a range of 3.6–5.9 years) purchased from commercial source (World Wide Primate, Miami, FL) were used. Upon receipt, NHPs were evaluated for general physical condition. Rectal swabs were screened for enteric pathogens and fecal examination was conducted for endoparasites. All animals were screened for exposure to simian immunodeficiency virus, simian retroviruses, simian T-cell leukemia virus type 1, herpes B virus, tuberculosis, shigella and tuberculosis prior to study initiation and quarantined for 35 days prior to the start of the experiment. Macaques were singly housed for the duration of the study. Animals were housed in stainless steel cages that meet requirements as set forth in the Animal Welfare Act (Public Law 99–198) and the Guide for the Care and Use of Laboratory Animals (8th Edition, Institute of Animal Resources, Commission on Life Sciences, National Research Council; National Academy Press; Washington D.C.; 2011). Animals were housed in environmentally monitored and ventilated rooms. Fluorescent lighting provided illumination approximately 12 h per day. Animals were observed a minimum of twice per day and clinical observations and stool consistency was noted on days following irradiation. The microbiome analysis reported in this research article was a secondary objective of the study. This study was conducted with the primary goal of analyzing the radiation injury in NHPs. The sample size for this NHP study, was based upon the goal to achieve statistically significant data for radiation injury. Samples from the cohorts included in the main study were used for gut microbiome analysis. Animals were continued with further monitoring for radiation injury natural history study after the fecal samples for microbiome analysis were collected (Day + 4 post-exposure).

### Irradiation

Dose rate measurements were based primarily on the alanine/EPR (electron paramagnetic resonance) system [20, 21], currently accepted as one of the most accurate methods for relatively high radiation doses and used for comparisons between national metrology institutions. The calibration curves (EMXmicro spectrometer, Bruker Corp., Billerica, MA, USA) used in dose measurements at Armed Forces Radiobiology Research Institute (AFRRI) are based on standard alanine calibration sets purchased from the US National Institute of Standards and Technology (NIST, Gaithersburg, MD, USA). The alanine dosimeters obtained from NIST had been calibrated in terms of absorbed dose to water using the US National Standard Radiation Sources. At AFRRI, identical alanine dosimeters were placed midline within NHP phantoms (Plexiglas cylinders 6.9, 10, 12.5 cm in diameter and 34.5 cm length) and irradiated to approximately 100 Gy. Measurement of their EPR signals using the calibration curve constructed with alanine dosimeters from NIST-provided dose rates to water in the core bodies of NHP. A small correction was subsequently applied for the difference in mass energy absorption coefficients between water and soft tissue. To deliver the precise dose, NHPs’ abdominal widths were measured with digital calipers. On the day of irradiation, food was withheld until after exposure. Macaques were sedated (Ketamine, 5 to 15 mg/kg, intramuscular) and placed in a positioning aide device (restraint box) prior to exposure. Radiation exposure was unilateral sequential (one half radiation dose from one side and then other half of the dose from other side). Each animal received a dose of 7.4 Gy at a dose rate of 0.6 Gy/min that took approximately 12.3 min. This was total-body irradiation. Since cancer patients receive organ specific partial-body exposure, their dose is higher and total time of exposure depends on total dose and dose rate that are different in different cases. After exposure, animals were allowed to recover from sedation and were returned to the holding facility.

### Fecal sample collection

Fecal samples were collected from each primate on days − 1, 1 and 4 post-exposure. Feces were collected directly from the cage with an applicator stick, placed in a 2 mL cryovial and frozen at − 70 °C. To ensure sterility, samples were collected first thing in the morning directly from the cage with a new sterile wooden or plastic applicator stick, placed in a 2 mL cryovial and frozen at − 70 °C or below. All of the samples used in this analysis were collected from animals that were treated identically and therefore blinding was not applicable to the in-life portion of the study. Sample details and animal ID# are shown in supplementary Table 1. The initial microbiome data generation (PCR at UAB microbiome core facility) was performed in a blinded fashion. However, the study design (sample/animal numbers for different time points) was revealed to the statistician for grouped analysis to perform statistical analysis.

### Fecal sample microbiome analysis

Microbial genomic DNA was isolated using a Fecal DNA isolation kit (Zymo Research) following the manufacturer’s instructions. Once the sample DNA was prepared, PCR was used with unique bar coded primers to amplify the variable region 4 (V4) region of the 16S rDNA gene to create an amplicon library from individual samples [22, 23]. The PCR product of ~ 255 bases from the V4 segment of the 16S rDNA gene was sequenced using single end reads using Illumina MiSeq [22]. None of the data was excluded. All the fecal microbiome data was included in the analysis. All of the samples used in this analysis were collected from animals that were treated identically and therefore randomization is not applicable. In the context of the larger study, animals were first grouped by age and then randomized based on weight using provantis. The animal study including the microbiome data analysis was conducted between April to October 2019.

To support the analysis of microbiome data, we have established an analytical pipeline based on the latest version of the QIIME tool suite [22, 24]. The first step in our analysis is to assess the quality of the raw data using FASTQC and then low quality data is filtered out using the FASTX toolset. The Ribosomal Database Program (RDP) classifier trained using the Greengenes (v13.8) 16S rRNA gene database was used to make taxonomic assignments for all OTUs at confidence threshold of 80% (0.8). The resulting OTU table included all OTUs, their taxonomic identification, and abundance information. OTUs whose average abundance was less than 0.005% were filtered out. OTUs were then grouped together to summarize taxon abundance at different hierarchical levels of classification (e.g. phylum, class, order, family, genus, and species). Alpha diversity (within sample diversity) was calculated using Shannon’s metrics as implemented in QIIME. Beta diversity (between sample diversity) among different samples was measured using three metrics; Bray Curtis (non-phylogeny based) (BC), weighted (W) and unweighted phylogeny based UNIFRAC (UW). Principal coordinates analysis (PCoA) was performed by QIIME to visualize the dissimilarity matrix (beta-diversity) between all the samples. 3D PCoA plots were generated using EMPEROR [22]. Distance between points (each sample shown as a point) reflects the differences in the composition between the samples.

#### Statistical analysis

For the microbiome analysis, samples were grouped by user defined variables and significant differences between groups determined by performing a PERMANOVA test on each of the beta diversity indices. A Kruskal–Wallis test was performed to identify key taxa whose changes in relative abundances between groups. These statistical tests were performed using tools within the QIIME package. Statistical analysis for Fig.2 (F/B Ratio) was conducted using Brown-Forsythe and Welch ANOVA test (GraphPad Prism 8.1.0, GraphPad Software, San Diego, California USA, www.graphpad.com).

## Results

NHPs (*N* = 19) were exposed to whole body 7.4 Gy cobalt-60 gamma-radiation (LD_70–80/60_) (Table 1), followed by fecal sample collection and monitoring for clinical signs of adverse effects with weekly body weight measurements. Fecal samples collected before (day − 1) and after (day + 1 and + 4) irradiation were subjected to 16S RNA gene sequencing.

**Table 1:** Study Design

Activity	Days procedures were conducted					
	−1	0	1	2	3	4
Irradiation		✔				
Clinical Monitoring		✔	✔	✔	✔	✔
Body weights	✔		✔			✔
Fecal collection	✔		✔			✔

### Alpha and beta diversity analysis pre and post-irradiation

**Fig. 1:**
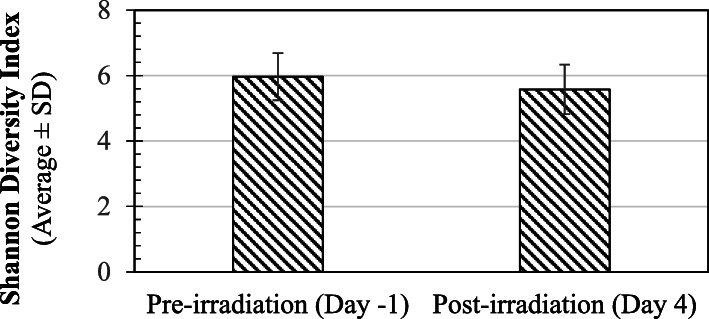
Alpha-Diversity in fecal microbiome at baseline and post-irradiation: Alpha Diversity was measured by the Shannon Diversity Index as described in the methods section. Shannon index (Average ± SD) for pre- and post-irradiation samples is shown on the Y-axis in the Fig. SD = Standard Deviation

441 to 560 OTUs were observed in the fecal samples at pre-irradiation (day − 1), whereas 381 to 578 OTUs were observed post-irradiation (day + 4). Diversity of microbes within the samples (Alpha Diversity) was measured by the Shannon Diversity Index, Chao1, and Simpson index (Supplementary Table 2). Shannon index was not significantly different between the pre- and post-irradiation sets of samples (5.96 ± 0.72 vs 5.58 ± 0.76 respectively) (Fig. 1). Similarly, other indices (Chao1 and Simpson index) also failed to show any significant difference (Supplementary Table 2).

To compare the diversity in the microbial composition between the fecal samples collected pre and post-irradiation, Beta diversity was measured using three metrics; Bray Curtis (non-phylogeny based) (BC), weighted (W) and unweighted phylogeny based UNIFRAC (UW). While the weighted UNIFRAC (W) analysis measures abundance of the microbiome, unweighted UNIFRAC (UW) does not account for the microbial abundance. As shown in Table 2, fecal samples immediately after irradiation (at day + 1) did not show a significant difference compared to pre-irradiation (day − 1) samples (*p* values not significant). In contrast, fecal samples at day + 4 post-irradiation showed a significant difference (p values < 0.05), suggesting significant differences in the microbe composition after irradiation, in a time dependent fashion.

**Table 2:** Beta Diversity in the fecal microbiome populations

Groups (Day-1 = baseline, Day + 1 and + 4 post-irradiation)	BC	UW	W
Day −1 vs Day + 1	0.139	0.628	0.506
Day − 1 vs Day + 4	0.006	0.012	0.005

BC = Non-phylogeny based Bray Curtis, W=Weighted phylogeny based UNIFRAC

*UW* Unweighted phylogeny based UNIFRAC

### Analysis of abundance at the phylum and genus levels

16S data base was used to analyze the microbial abundance at the Phylum and Genus levels, shown in Table 3. As there was no significant change in the beta diversity immediately after the irradiation (day + 1 post-irradiation) (Table 2), analysis was limited to samples collected later (day + 4) in comparison to pre-irradiation (day − 1) samples.

**Table 3:** Altered fecal microbiome profile post-Irradiation

Level	OTU	P	FDR P	Bonferroni P	Prevalence Day − 1	Prevalence Day + 4	Fold Change (Day + 4/Day − 1)
Phylum	Bacteroidetes	0.008	0.125	0.125	39%	52%	1.32
	Firmicutes	0.033	0.209	0.467	46%	39%	0.85
Genus	[Prevotella]	0.000006	0.0006	0.0007	0.44%	2.05%	4.69
	Acinetobacter	0.000021	0.001	0.003	0.17%	0.00%	0.01
	Aerococcus	0.000097	0.003	0.011	0.23%	0.02%	0.10
	Actinobacillus	0.001	0.018	0.124	0.01%	0.11%	9.88
	Veillonella	0.001	0.018	0.147	0.01%	0.11%	11.16
	Bacteroides	0.002	0.027	0.247	0.04%	0.11%	2.65

*OTU* Operational Taxonomic Unit, *FDR* False Discovery Rate, p = p value (for statistical significance), * Paraprevotellaceae family

**Fig. 2:**
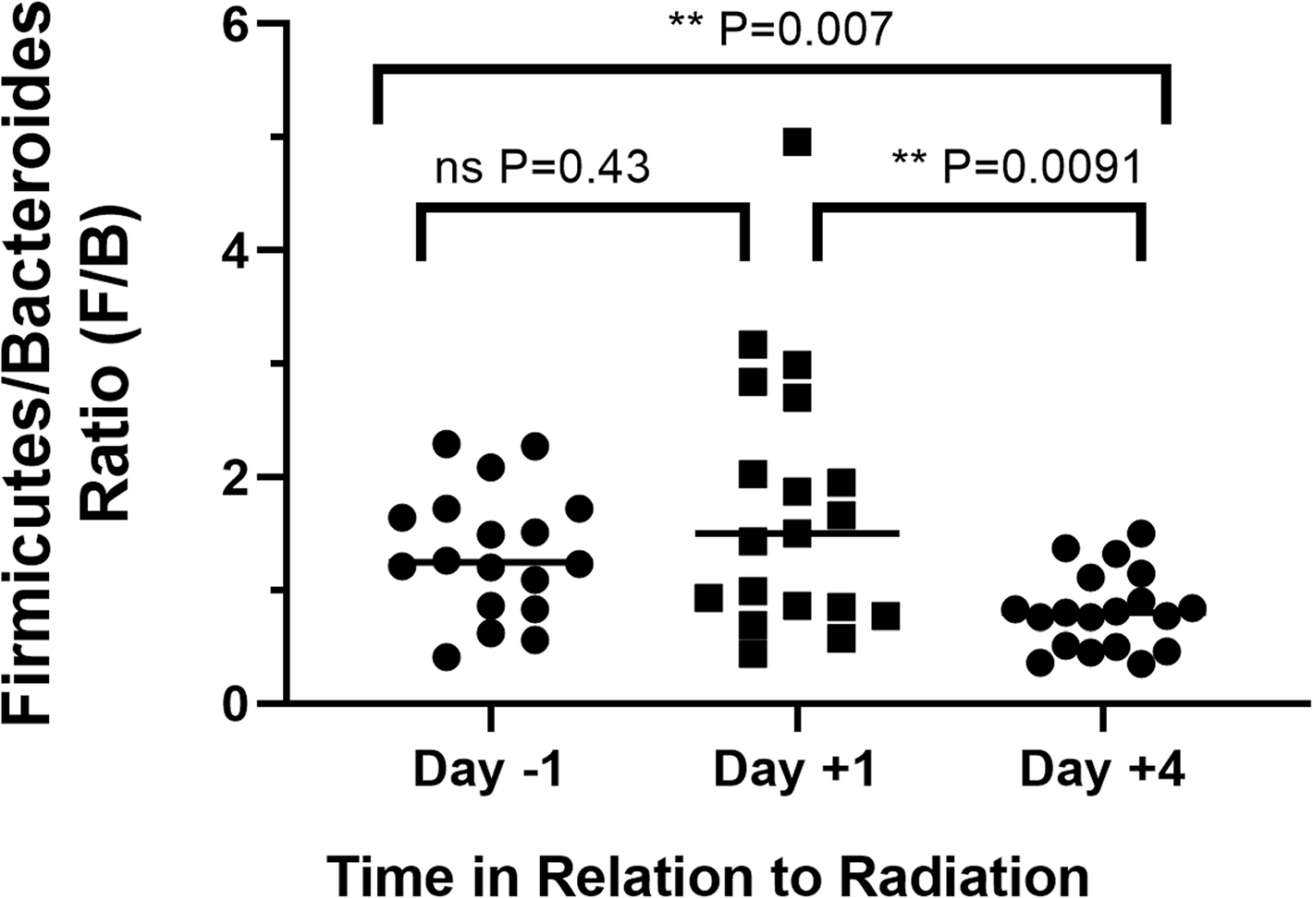
Firmicutes/Bacteroidetes (F/B) Ratio at baseline and post-irradiation: Prevalence of Firmicutes and Bacteroidetes phylum was measured in the fecal samples of each animal collected at baseline and different time points post-irradiation. Ratio of % Prevalence of Firmicutes and Bacteroidetes (F/B) is shown on the Y-axis. ns = not-significant, *p* > 0.05, P = *p* < 0.5

Before irradiation (day − 1), Bacteriodetes and Firmicutes were the major phyla observed (39 to 46%). Other minor phyla such as Spirochaetes (5.6%), Verrucomicobria (1.2%), and less than 1% of other phyla (Euryarcheaota, Lentisphaerae, Tenericutes) were observed. As noted elsewhere [12, 25], baseline gut microbiota was dominated by members of the *Prevotella* (30.5%) genus (data not shown). However, after irradiation (Day + 4) the prevalence of Bacteroidetes phylum increased from 39 to 52% (1.3-fold increase), whereas Firmicutes decreased from 46 to 39% (0.84-fold decrease), resulting in a significant decrease in the Firmicutes/Bacteriodes (F/B) ratio from 1.2 at pre-irradiation (day − 1) to 0.75 at post-irradiation (day + 4) (*p* = 0.007) (Fig. 2). In contrast, there was no significant difference (ns, *P* = 0.43) between day − 1 and day + 1 samples. Day + 4 samples also showed significantly lower F/B ratio (*P* = 0.0091) compared to day + 1.

Representation of four genera, *Prevotella (Paraprevotellaceaea family)*, *Actinobacillus*, *Veillonella* and *Bacteroides,* was found to be increased between 2 and 11 fold, whereas representation of two genera, − *Acinetobacter* and *Aerococcus,* decreased by 10 and 100 fold, respectively, at day + 4 post-irradiation (Table 3). Considering the prevalence before irradiation (more than 0.1%), three differences at the genus levels at day + 4 post-irradiation were noteworthy. These include, increased *Prevotella (Paraprevotellaceaea family)* (more than 4-fold increase) and decreased *Acinetobacter* and *Aerococcus* (more than 10 to 100-fold decreases, respectively).

### Microbial changes in animals with diarrhea post-irradiation

Diarrhea was observed in some animals in a time dependent manner as a consequence of irradiation. None of the animals had shown diarrhea pre-irradiation, and day + 1 and + 2 post-irradiation, whereas 10 out of 19 animals (52.6%) showed diarrhea at day + 4 post-irradiation. By day 6, 79% of the animals came down with diarrhea. As we wanted to study the early microbial changes associated with diarrhea, we focused on animals with diarrhea at day + 4. Comparison of the microbiome of animals with and without diarrhea was performed to understand the microbial differences and visualize similarities or dissimilarities of gut microbiome using Principal Coordinates Analysis (PCoA). In this analysis, points that are closer together represent microbial communities that are more similar in composition.

**Fig. 3:**
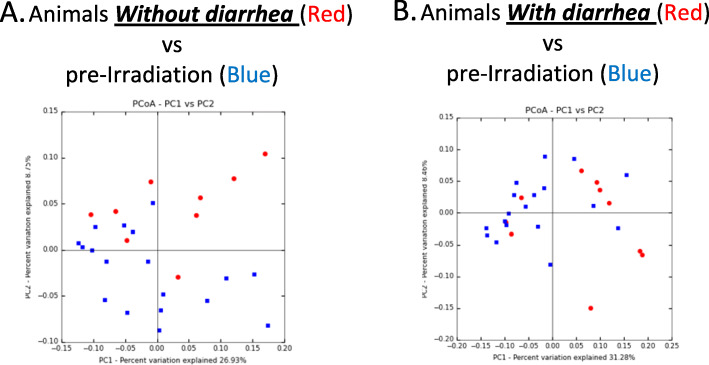
PCoA plots of gut microbiome from animals with and without diarrhea at day + 4 post-irradiation compared to baseline: Gut microbiome analysis (unweighted UNIFRAC) of animals without diarrhea at day + 4 (**a**) and diarrhea **(b**) in comparison shown by the PCoA plot analysis. Baseline samples are shown in blue. Day + 4 post-irradiation samples are shown as red. Numbers (26.93) and (31.28) on PC1 represent the maximum percentage variation explained on the designated axis. PCoA = Principle Coordinates Analysis

PCoA plot analysis for the gut microbiome (unweighted UNIFRAC) of animals with and without diarrhea at day + 4 post-irradiation in comparison to baseline was performed (Fig. 3). Distance between points (each point represents a sample) depicts compositional differences. The samples are colored by baseline (blue) vs animals with diarrhea or without diarrhea (red), showing a difference in the microbial community composition between irradiated (red) and baseline (blue).

The clustering of samples from animals without diarrhea post-irradiation (red dots) were distinct from the baseline samples (blue dots) suggesting significant changes in the microbiome population after irradiation at day + 4, in animals without diarrhea (Fig. 3a Table 2) compared to baseline. Interestingly, we did not observe significant changes in the microbiome population after irradiation at day + 4 in animals with diarrhea (Fig. 3b), compared to baseline (Table 2). Detailed analysis of microbiome changes in animals with diarrhea suggested three significant differences in these animals (Table 4). *Lactobacillus reuteri* was significantly increased (17 fold) in prevalence (0.06 to 1.09%) in animals with diarrhea. Similarly, two genera, *Dialister (14.9 fold)* and *Veillonella* (32.9 fold) were also found to be increased in animals with diarrhea compared to animals without diarrhea.

**Table 4:** Altered fecal microbiome observed in animals with Diarrhea Post-Irradiation

OTU	P	FDR P	Bonferroni P	Prevalence in Animals without Diarrhea	Prevalence in Animals With Diarrhea	Fold Increase in diarrhea animals
g_Dialister	0.012	0.731	1	0.01%	0.15%	14.9
g_Veillonella	0.012	0.731	1	0.01%	0.16%	32.9
g_Lactobacillus;s_reuteri	0.017	0.731	1	0.06%	1.09%	17.8

*g* Genus, *s* species, *OTU* Operational Taxonomic Unit, *FDR* False Discovery Rate, p = *p* value (for statistical significance),

### Biomarkers for radiation-induced diarrhea

Diarrhea is a significant side effect of radiation injury [11]. It would be beneficial to prospectively identify subjects who might be prone to radiation induced diarrhea. This would aid in the development of appropriate therapy to prevent radiation-induced diarrhea. To identify microbial biomarkers for susceptibility to radiation-induced diarrhea, fecal samples of animals at pre-irradiation (day − 1) with and without diarrhea symptom post-irradiation (day + 4) were compared (Table 5). Before irradiation (day − 1), two phylum of bacteria were associated with diarrhea at day + 4. Animals with diarrhea had significantly lower levels of *Lentisphaere* and *Verrucomicrobia* phyla and significantly lower levels of *Bacteroides* genus before irradiation. These differences show the potential association between the prevalence of microbiomes and differential susceptibility to radiation-induced diarrhea.

**Table 5:** Biomarker at baseline predictive of Diarrhea post-irradiation (Day + 4)

Level	OTU	P	FDR P	Bonferroni P	Prevalence at baseline in Animals without Diarrhea at day + 4	Prevalence at baseline in Animals with Diarrhea at day + 4	Fold Change (Diarrhea/Non-Diarrhea)
Phylum	Verrucomicrobia	0.009	0.104	0.128	1.68%	0.45%	−3.7 fold
	Lentisphaerae	0.014	0.104	0.209	0.69%	0.08%	−8.6 fold
Genus	Bacteroides	0.002	0.266	0.266	0.07%	0.02%	−3.5 fold

*g* Genus, *s* species, *OTU* Operational Taxonomic Unit, *FDR* False Discovery Rate, *p* p value (for statistical significance),

## Discussion

Despite the significant clinical incidence of intestinal radiation injury in cancer patients, effective therapies to address intestinal radiation injury are not available. NHPs seem to reproduce several aspects of acute radiation syndrome (ARS) observed in humans (Reviewed in [16]). In light of similarity to physiological responses to irradiation in humans, evolutionary relatedness, similarity in organ structure; GI symptoms and metabolism to humans, NHPs are considered to be the benchmark animal model for studying ARS. One of the clinical manifestations of ARS include gastrointestinal effects (at exposure levels of more than 6 Gy) [26]. Though probiotics are shown to exert positive effect on the ARS [14], specific and effective treatments are unavailable.

In the current study, we used NHP model of ARS and evaluated the gut microbial changes at early time points post-irradiation. We included only males in our study to avoid the effects of female hormone on the radiation injury. Radiation injury was measured by evaluating complete blood cell counts at regular intervals after irradiation. We observed typical depletion of blood cells indicative of radiation injury as reported earlier [27]. Longitudinal microbiome analysis (using the baseline of animals before the irradiation) was used in the current study, as a recent paper has shown the enhanced statistical power provided by the longitudinal studies compared to larger cross sectional studies [28].

In our study, we did not observe significant differences in the alpha diversity (number of species) between pre (day − 1) and post-irradiation (day + 4), which might be due to the short period of post-irradiation (4 days), resulting in altered number of microbiota in the individual species, without affecting the number of microbial species. Alpha diversity might be affected with longer duration of radiation exposure, but not with the short term radiation exposure used in the current study. Microbiome analysis at day 4 post-irradiation, might not be sufficient to affect alpha diversity. Though differences in alpha diversity were not observed, significant changes in beta diversity after irradiation consistent with the previous studies [12, 18] were noticed. The observed change in beta diversity was further strengthened with the significant decrease in the Firmicutes/Bacteriodes (F/B) ratio (from 1.2 at pre-irradiation at day − 1 to 0.75 at post-irradiation day + 4), as Firmicutes/Bacteriodes (F/B) ratio is considered to be a significant indicator of gut microbiota composition [29, 30]. Interestingly significant increase in Bacteroidetes phylum at day + 4 post-irradiation observed in our study, differs from couple of previous studies [12, 18]. These previous reports had shown either non-significant increase in Bacteroidetes in Chinese rhesus macaques after 3-days post-irradiation with 6.8 Gy radiation [12] or no change in Bacteroidetes with 6.8/7.2/7.7 Gy radiation in Chinese rhesus macaques after 3-days post-irradiation [18].

Significant changes in specific microbes *[Actinobacillus, Bacteroides, Prevotella (Para-prevotellaceae family), Veillonella*, *Acinetobacter* and *Aerococcus]* were observed in the current study. Changes in the levels of *Actinobacteria*, *Veillonella* and *Bacteriodes* genera post-irradiation observed in our study are similar to the previous reports [12, 18]. However, in contrast to these previous reports [12, 18], our study failed to observe significant changes in number of other genera (*Spirochetes*, *Lactobacillus*, *Streptococcus*, *Treponema*, *Helicobacteria*, *Parabacteroides*, *Collinella* and *Prevotella*). This discrepancy might be due to differences in study cohorts and slight difference in study time points for microbial analysis (day+ 4 post-irradiation in our study vs day+ 3 post-irradiation in the reports). This disagreement also underscores the effect of different cohorts and time point post-irradiation selected for analysis of gut microbiota. Results of our study suggest that biomarkers of irradiation intensity as mentioned in the Carbonero et al. [18], may not be universal and might depend upon several other factors such as profile of cohorts and diets.

Fecal microbiome analysis also revealed significantly elevated *Lactobacillus reuteri* in 52 % (10/19) of the irradiated animals which showed diarrhea at day 4 post-irradiation*.* Increased levels of *Lactobacillus reuteri* in animals with diarrhea might be due to the body’s early protective response against diarrhea, as *Lactobacillus reuteri* is associated with intestinal health and has shown to improve the gut health and decrease the duration of diarrhea [31]. Elevated levels of *Dialister* and *Veillonella* observed in our study is in line with the previous literature reports where *Dialister* and *Veillonella* are associated with radiation-induced diarrhea [32]. Radiation enteritis [33, 34] can be caused by several pathological changes such as progressive obliterative endarteritis, submucosal fibrosis manifested by stricturing, fistulae, local abscesses, perforation, and bleeding. Though such “radiation enteritis associated pathological changes” might cause diarrhea, changes in the gut microbiota composition at an early time point post-irradiation (day + 4), might act as an initial trigger for diarrhea.

Our results also suggest that animals with diarrhea at day 4 post-irradiation, revealed lower levels of *Lentisphaerae* and *Verrucomicrobioa* phyla and *Bacteroides* genus at baseline (before irradiation) shedding some light on the possible role of these bacteria in maintaining intestinal health. Association of significantly lower levels of *Lentisphaere* and *Verrucomicrobia* phyla and *Bacteroides* genus at baseline in animals that showed diarrhea after irradiation is interesting and warrants further detailed investigation. In light of this observation, it remains to be seen if the radiation-induced diarrhea can be reversed by use of probiotics containing these bacteria and modify the kinetics of restoration of bowel health. Our current study had also some limitations. Our sample analysis was limited to the early time points (up to day + 4), as we wanted to focus on the early primary changes and not a consequence of other effects of irradiation on the body (secondary events). Our observations are limited to fecal microbiome analysis. Additional metabolomics studies of the fecal and serum samples can add value to these findings.

Though the previous studies [12, 18] had shown changes in microbiome in the irradiated NHPs, our study is unique in two aspects; 1) demonstration of association between diarrhea and specific microbiome changes, and 2) microbial biomarker at baseline which might be suggestive of radiation-induced diarrhea. However, the false discovery analysis was not significant which suggests the need for larger sample size to confirm this finding.

## Conclusions

We found that whole-body irradiation altered the diversity of the microbiome and relative representation of several key genera or species. Irradiation-induced diarrhea was observed in roughly half of the animals by day 4 post-exposure, which appeared to be associated with an increase in *Lactobacillus reuteri,* a species usually associated with protection from diarrhea. This study demonstrates that exposure to ionizing radiation can have a significant impact on the composition of the gut microbiome, and particular species may be more susceptible to irradiation, while others may predict harmful side effects. Overall, our study demonstrates significant microbial alterations in the NHP model of radiation injury and provides a valuable insight into early changes in the gut microbiome after radiation exposure. Data observed in our study also paves the way for testing the effect of different antibiotics on the radiation-induced gut microbial changes and the associated diarrhea. Additional studies are needed to understand the impact of microbial changes on the pathophysiology of the animals and the effect of probiotics/antibiotics in preventing the radiation-induced diarrhea. NHP model of radiation injury can serve as a valuable tool to identify microbiome biomarkers of radiation exposure and assist in developing effective therapeutic intervention to mitigate the radiation injury. Understanding the pathophysiology of radiation injury and radiation-induced diarrhea can help in the development of new drugs and preventive measures (probiotics). Such efforts will be a great benefit for society as it can enhance the quality of life of cancer patients subjected to irradiation.

## Supplementary Information

**Additional file 1: Supplementary Table 1**: Sample details and Animal ID. **Supplementary Table 2**: Alpha Diversity.

## Data Availability

The data and sequences of the material in the manuscript is available at the following link https://data.genome.uab.edu/ccts/bmi/microbiome/result2019/M220/PrinceTuffourSRI_analysis/ANALYSIS/microbiome_report.html

## Abbreviations

NHP: Non human primates
DNA: Deoxyribo nucleic acid
GI-tract: Gastro-intestinal tract
OTU: Operational Taxonomic Unit
F/B: Firmicutes/Bacteriodes
ARS: Acute Radiation Syndrome
FDR: False Discovery Rate
