# Supplementary material for: Changes in the gut microbiome community of nonhuman primates following radiation injury
Source: BMC Microbiol. 2021 Mar 29;21:93. doi: 10.1186/s12866-021-02146-w (PMC8008626; doi:10.1186/s12866-021-02146-w)
Supplement: Supplementary file 1 — Additional file 1: Supplementary Table 1: Sample details and Animal ID. Supplementary Table 2: Alpha Diversity. [file 12866_2021_2146_MOESM1_ESM.docx]

**Supplementary Materials**

**Supplementary Table 1: Sample details and Animal ID**

| **Sample name** | **Animal ID#** | **Day** | **Sample name** | **Animal ID#** | **Day** | **Sample name** |  | **Animal ID#** | **Day** |
| --- | --- | --- | --- | --- | --- | --- | --- | --- | --- |
| 137270 | 5753 | -1 | 137290 | 5767 | 4 | 139316 |  | 5764 | 1 |
| 137271 | 5759 | -1 | 137291 | 5756 | 4 | 139317 |  | 5760 | 1 |
| 137272 | 5767 | -1 | 137292 | 5761 | 4 | 139318 |  | 5776 | 1 |
| 137273 | 5756 | -1 | 137293 | 5768 | 4 | 139319 |  | 5763 | 1 |
| 137274 | 5761 | -1 | 137294 | 5770 | 4 | 139320 |  | 5754 | 1 |
| 137275 | 5768 | -1 | 137295 | 5762 | 4 | 139321 |  | 5771 | 1 |
| 137276 | 5770 | -1 | 137296 | 5773 | 4 | 139322 |  | 5758 | 1 |
| 137277 | 5762 | -1 | 139303 | 5779 | -1 | 139323 |  | 5779 | 4 |
| 137278 | 5773 | -1 | 139304 | 5757 | -1 | 139324 |  | 5757 | 4 |
| 137279 | 5753 | 1 | 139305 | 5747 | -1 | 139325 |  | 5747 | 4 |
| 137280 | 5759 | 1 | 139306 | 5764 | -1 | 139326 |  | 5764 | 4 |
| 137281 | 5767 | 1 | 139307 | 5760 | -1 | 139327 |  | 5760 | 4 |
| 137282 | 5756 | 1 | 139308 | 5776 | -1 | 139328 |  | 5776 | 4 |
| 137283 | 5761 | 1 | 139309 | 5763 | -1 | 139329 |  | 5763 | 4 |
| 137284 | 5768 | 1 | 139310 | 5754 | -1 | 139330 |  | 5754 | 4 |
| 137285 | 5770 | 1 | 139311 | 5771 | -1 | 139331 |  | 5771 | 4 |
| 137286 | 5762 | 1 | 139312 | 5758 | -1 | 139332 |  | 5758 | 4 |
| 137287 | 5773 | 1 | 139313 | 5779 | 1 |  |  |  |  |
| 137288 | 5753 | 4 | 139314 | 5757 | 1 |  |  |  |  |
| 137289 | 5759 | 4 | 139315 | 5747 | 1 |  |  |  |  |

**Supplementary Table 2: Alpha Diversity**
